# Establishing Local Diagnostic Reference Levels (DRLs) for Adult Computed Tomography in Emirates Health Services Hospitals: A Multicenter Dose Survey

**DOI:** 10.3390/diagnostics16091353

**Published:** 2026-04-30

**Authors:** Amina Aljasmi, Sheikha Almsafri, Suhaib Alameen, Hatem Ghonim, Maryam Alhajri, Amna Alshamsi, Sherif Hani Elkelsh, Mohammed Abuzaid

**Affiliations:** 1 Radiology Department, Emirates Health Services, Dubai P.O. Box 70225, United Arab Emirates; 2 Radiation Protection Center, Radiology Department, Emirates Health Services, Dubai P.O. Box 70225, United Arab Emirates; 3 Unison Capital Investment LLC, Dubai Hills Estate, Dubai P.O. Box 66212, United Arab Emirates; 4 Department of Medical Diagnostic Imaging, College of Health Sciences, University of Sharjah, Sharjah P.O. Box 27272, United Arab Emirates

**Keywords:** Diagnostic Reference Levels (DRLs), Computed Tomography (CT), CTDIvol, Dose-Length Product (DLP), radiation dose optimization, patient safety

## Abstract

**Objectives**: This study aimed to establish local Diagnostic Reference Levels (DRLs) for adult computed tomography (CT) across Emirates Health Services (EHS) hospitals in the United Arab Emirates. **Methods**: A retrospective multicenter survey included 1257 adult patients from seven EHS hospitals who underwent six routine CT protocols: head without contrast (*n* = 375), chest without contrast (*n* = 403), chest with contrast (*n* = 50), abdomen–pelvis without contrast (*n* = 204), abdomen–pelvis with contrast (*n* = 164), and chest–abdomen–pelvis (*n* = 61). Only single-phase, standard-range examinations were included. Examinations with major protocol deviations, extended scan ranges, or manual exposure overrides were excluded. CTDIvol and DLP were extracted from DICOM dose reports and reviewed against protocol definitions and scanner dose documentation. Local DRLs were defined as the 75th percentile of the dose distribution for each protocol, and median values were reported as achievable dose indicators. **Results**: Inter-hospital variability was observed across all protocols, particularly for abdomen–pelvis and chest–abdomen–pelvis examinations. The proposed DLP-based local DRLs (mGy·cm) were: head without contrast, 1179.6; chest without contrast, 425.0; chest with contrast, 1238.0; abdomen–pelvis without contrast, 637.2; abdomen–pelvis with contrast, 1269.9; and chest–abdomen–pelvis, 1411.5. Median values indicated achievable doses below the 75th percentile for all protocols. Compared with selected international studies, abdomen–pelvis doses were broadly comparable, whereas head and chest doses were somewhat higher. **Conclusions**: This study provides a coordinated multicenter baseline for adult CT local DRLs across EHS hospitals. The findings support protocol harmonization, scan-length optimization, targeted staff training, and integration with dose-monitoring systems to strengthen CT dose optimization and patient safety and to inform future updates of UAE national DRLs.

## 1. Introduction

Computed tomography (CT) has revolutionized diagnostic radiology by providing detailed cross-sectional images that significantly enhance diagnostic accuracy [1,2]. CT scans offer high-contrast resolution, superior tissue differentiation, and multiplanar reconstructions, aiding in the diagnosis of a wide range of medical conditions [3,4]. The use of iodinated contrast media further enhances tissue differentiation in CT examinations [5]. Recent advancements, such as four-dimensional CT, cone-beam CT, and dual-energy CT, have expanded CT utility in musculoskeletal imaging, trauma assessment, and disease characterization by improving spatial resolution and quantitative capabilities. Despite the relatively high radiation exposure associated with CT, its benefits, including rapid scanning, objective density measurements, and the ability to assess contrast enhancement, make it indispensable in modern diagnostic practice.

Awareness of the risks associated with CT examinations is crucial because ionizing radiation exposure may increase the lifetime risk of cancer. Studies have suggested associations between CT exposure and thyroid cancer, leukemia, and other malignancies, especially in sensitive age groups [6,7,8,9,10]. Therefore, CT protocols must be optimized to minimize radiation dose while preserving diagnostic image quality. Healthcare providers and radiology staff should be educated about radiation risks and dose optimization strategies to ensure safe and effective CT practice.

Establishing Diagnostic Reference Levels (DRLs) is a key component of radiation protection optimization. DRLs provide practical benchmarks for dose auditing, identifying unusually high exposures, and supporting quality improvement. Importantly, DRLs are not dose limits; rather, they are tools for optimization. They are typically defined using the 75th percentile of dose distributions for a given examination type and patient population [11,12].

In the United Arab Emirates, national Diagnostic Reference Levels (NDRLs) were first introduced in 2017 and endorsed by the Federal Authority for Nuclear Regulation (FANR). These initial benchmarks included CT examinations such as brain, brain with contrast, chest, and abdomen–pelvis. However, these early NDRLs were based mainly on DLP values and were intended as a starting point for further facility-level and national dose surveys. Subsequent work in the UAE has shown the value of more detailed, protocol-specific dose assessments [13,14,15]. In this context, Emirates Health Services (EHS) has prioritized DRL implementation to strengthen radiation safety, standardize CT practice, and support ongoing national dose optimization efforts.

This study was designed to establish local DRLs for common adult CT protocols across EHS hospitals and to provide a practical benchmark for dose monitoring and protocol optimization. In addition, the survey provides an updated overview of CT equipment and acquisition practices across participating centers.

## 2. Materials and Methods

### 2.1. Study Design and Setting

This retrospective, multicenter cross-sectional study was conducted across seven Emirates Health Services (EHS) hospitals located in five Emirates between January 2023 and November 2024. All participating facilities operated multi-detector CT scanners (16-, 64-, and 128-slice systems) under established quality assurance programs and regulatory oversight by the Federal Authority for Nuclear Regulation (FANR).

The study was designed in accordance with the methodological framework detailed in ICRP Publication 135 for Diagnostic Reference Level (DRL) surveys, including protocol standardization, adult-weight-based standardization, and quality assurance measures to ensure comparability across institutions.

Table 1 summarizes the CT scanner characteristics of the participating Emirates Health Services hospitals. All scanners were manufactured by GE Healthcare, Chicago, IL, USA, and included the Discovery HD 750 at Fujairah Hospital, Revolution at Dibba Hospital, Brivo CT385 at Masafi Hospital, Revolution Maxima at Shaam Hospital, Optima CT660 SGT1700 at Abdulla bin Omran Hospital, Revolution at Umm Al Quwain Hospital, and Revolution at Al Dhaid Hospital. The detector row configuration ranged from 16 to 128 slices, and the installation dates ranged from 2015 to 2022.

### 2.2. Patient Selection and Standardization

Adult patients (≥18 years) undergoing routine, single-phase CT examinations were included. Only standard-range scans performed for routine clinical indications were considered to ensure methodological consistency across sites.

For adult DRL surveys, patient standardization was achieved by restricting inclusion to individuals within a representative weight range to approximate the reference adult population. In accordance with international practice, particularly ICRP 135 guidance, a weight range of 50–90 kg was applied to achieve an approximate mean reference weight of 70 kg. This approach reduced dose variability related to extremes of body habitus and improved comparability across hospitals while maintaining routine clinical relevance.

### 2.3. Exclusion Criteria Included

Six high-frequency adult CT protocols were analyzed, including head without contrast, chest without contrast, chest with contrast, abdomen–pelvis without contrast, abdomen–pelvis with contrast, and chest–abdomen–pelvis examinations. Examinations were excluded if they involved multiphase acquisitions, extended or non-standard scan ranges, trauma or interventional protocols, follow-up or repeat series during the same visit, pediatric patients younger than 18 years, cases outside the predefined adult weight range, manual exposure overrides, non-standard kV or mAs settings, or protocol misclassification, ensuring data consistency and methodological standardization across all included CT examinations.

### 2.4. CT Protocol Verification and Quality Assurance

Protocol parameters, including kVp, mAs, pitch, and rotation time, were extracted from patient-level acquisition data rather than preset scanner templates. All hospitals routinely used automatic tube current modulation and iterative reconstruction, while automatic kV selection was available on some scanners, depending on the manufacturer and installation year.

Protocol verification was performed using DICOM metadata, scanner dose reports, and examination naming conventions. Final protocol classification was confirmed by dual review from a radiologist and a CT technologist to reduce mislabeling and ensure accurate grouping of examinations.

CT scanners at participating hospitals were subject to routine quality assurance, including dose output checks and standard calibration procedures. However, all centers followed EHS quality assurance policies; the exact availability and implementation of dose-saving technologies varied by scanner model and installation year.

### 2.5. Radiation Dose Assessment

For each examination, CTDIvol (mGy) and total DLP (mGy·cm) were extracted from DICOM dose reports. Scanner-reported CTDIvol values were interpreted according to the appropriate reference phantom, using the 16 cm phantom for head examinations and the 32 cm phantom for body examinations.

Examinations with deviations from standard acquisition protocols, such as extended scan length, manual override of automatic exposure control, or non-standard kV/mAs settings, were excluded from DRL calculation to maintain methodological consistency.

Local DRLs were defined as the 75th percentile of the dose distribution for each protocol. Median values were reported as achievable dose indicators. In accordance with international guidance, DRLs were used as reference levels for investigation and optimization, not as mandatory dose limits.

### 2.6. Image Quality Assessment

All CT examinations included in the analysis were reviewed during routine clinical workflow by the responsible CT technologist at the time of acquisition and subsequently interpreted by a consultant radiologist at the respective hospital. Image quality was considered diagnostically acceptable in all included examinations. No examinations were excluded because of inadequate image quality. This ensured that the resulting local DRLs reflected routine diagnostic practice while maintaining acceptable image quality across centers.

### 2.7. Statistical Analysis

Data were organized using Microsoft Excel and analyzed with SPSS version 23 (IBM Corp., Armonk, NY, USA). Descriptive statistics included mean, standard deviation, median, 75th percentile, range, and interquartile range for CTDIvol and DLP.

Inter-hospital variability and high-dose outliers were examined descriptively. Inferential hypothesis testing was not performed, as the primary objective was the establishment of local DRLs rather than statistical comparison between hospitals. Established DRLs were compared with selected national and international reference values to interpret findings within regional and global practice.

### 2.8. Ethical Approval

Ethical approval was obtained from the MOHAP Research Ethics Committee (Reference: MOHAP/REC-82/2024). The requirement for informed consent was waived because the analysis was retrospective and involved anonymized dose data.

## 3. Results

Data from 1257 adult CT examinations were analyzed across seven hospitals using 16- to 128-slice CT scanners. The included cohort was restricted to adult patients within the predefined standard weight range to improve comparability across centers. Considerable inter-hospital variability was observed for all protocols, particularly for chest and combined examinations.

Median and 75th percentile DLP values increased systematically from single-region to multi-region scans. The highest proposed local DRL was observed for chest–abdomen–pelvis examinations, while the lowest was for chest without contrast. The widest dose ranges were observed for abdomen–pelvis and combined chest–abdomen–pelvis protocols, reflecting variations in scan length and protocol selection.

Chests with contrast showed substantially higher CTDIvol and DLP values than those without contrast, indicating that contrast-enhanced chest protocols were more variable and likely included broader clinical indications or longer acquisition ranges in some centers. This pattern was consistent with the higher 75th percentile DLP observed for the chest with contrast.

Inter-hospital variability was most pronounced in abdomen–pelvis and combined protocols, suggesting that local practice differences remained a major determinant of dose. These findings support the need for protocol harmonization and standardization of scan length.

Table 2 summarizes the demographic characteristics of patients who underwent different CT examinations, providing detailed age, height, weight, and body mass index (BMI) data for each type of scan, kilovoltage peak (kVp), milliampere-seconds (mAs), rotation time (T rotation), and pitch, for different CT examinations. The values are presented as means ± standard deviations (Mean ± SD) along with their respective ranges. The demographic characteristics of patients undergoing various CT examinations show a wide age range, with most having a mean age between 38 and 54 years. Patient height averages around 163 to 167 cm, and weight averages between 71 and 74 kg across different scans. These common characteristics of patients in the study provide a baseline for understanding the variability in CT imaging practices. In parallel, Table 3 presents key CT protocol parameters and dose indices. Kilovoltage (kV) is set between 116 and 120, while milliampere-seconds (mAs) show variation according to examination type, from 92.9 in chest with contrast to 165.9 in head without contrast. The average rotation time (Trot) ranges from 0.6 to 0.75 s, and pitch values span 0.5 to 1.17. Notable variation is also observed in CTDIvol and DLP, reflecting the differing radiation demands across scan types. CTDIvol ranges from 7.4 mGy in chest with contrast to 51.3 mGy in head without contrast, whereas DLP spans from 356.8 mGy·cm in chest without contrast to 1094.7 mGy·cm in chest, abdomen, and pelvis examinations. This integrated showed the importance of customized protocol optimization to ensure diagnostic efficacy while minimizing patient exposure.

The proposed Local DRLs, defined as the 75th percentile of the DLP distribution, are represented in Table 4. A hospital-level summary of median, 75th percentile, and range values should be provided in a supplementary table, and box plots should be added to more clearly visualize inter-institutional variability.

## 4. Discussion

This multicenter survey established coordinated local DRLs for common adult CT examinations across seven Emirates Health Services (EHS) hospitals, based on data from 1257 patients. DRLs were calculated using CTDIvol and DLP metrics at the 75th percentile, providing a baseline for dose tracking and optimization. The 75th percentile DLP-based DRLs for adult CT examinations across EHS hospitals were as follows: Chest without contrast: 425.0 mGy·cm, Chest with contrast: 1238.0 mGy·cm, Head without contrast: 1179.6 mGy·cm, Abdomen–pelvis without contrast: 637.2 mGy·cm, Abdomen–pelvis with contrast: 1269.9 mGy·cm, and Chest–abdomen–pelvis: 1411.5 mGy·cm.

**Figure 1:**
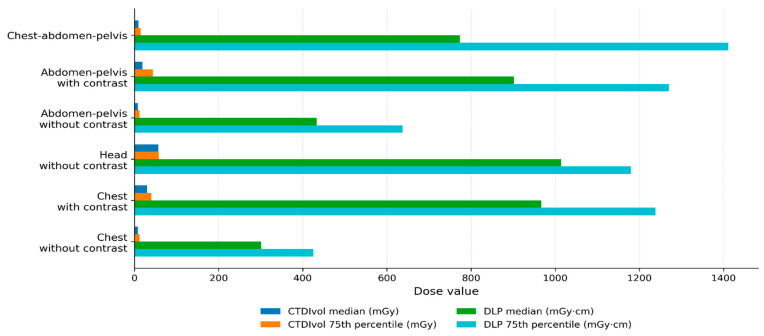
Local CT dose metrics and technical parameters by examination protocol.

As illustrated in Figure 1 and Table 3, the distribution of CTDIvol and DLP across examination protocols demonstrates substantial dose variation depending on anatomical region, scan length, and the use of contrast enhancement. Chest without contrast consistently showed the lowest dose values. In contrast, chest–abdomen–pelvis and contrast–enhanced abdomen–pelvis examinations exhibited the highest DLP values, largely due to extended scan coverage and increased protocol complexity. Additionally, the 75th percentile values exceeded median values across all protocols, indicating a right-skewed dose distribution and highlighting higher-dose examinations associated with scan length variability, patient size differences, and exposure settings. Variation among hospitals was particularly notable for abdomen–pelvis and chest–abdomen–pelvis protocols, reflecting differences in scan length, contrast phase selection, and local protocol implementation rather than scanner model alone. Although technologies such as automatic tube current modulation, iterative reconstruction, and automatic kV control support dose optimization, differences in scanner models and installation years limited uniform implementation, emphasizing the need for protocol harmonization, standardized scan coverage, and regular staff training.

Variation among hospitals was especially notable for abdomen–pelvis and chest–abdomen–pelvis protocols. This variability most likely reflects differences in scan length, contrast phase selection, local protocol implementation, and examination indication rather than scanner model alone. These findings reinforce the importance of consistent protocol design, standardized scan coverage, and regular staff training. Although automatic tube current modulation, iterative reconstruction, and automatic kV control can reduce dose, their availability and implementation varied across hospitals because scanner models and installation years differed. Therefore, these technologies support optimization but do not eliminate inter-hospital variability.

The higher DLP observed for the chest with contrast compared with the chest without contrast may reflect broader clinical indications, additional coverage in some cases, or differences in reconstruction or exposure settings. Contrast-enhanced chest studies are often performed in more complex clinical scenarios, which may require longer scan coverage or tailored acquisition parameters. This may explain the higher CTDIvol and DLP values observed in this protocol.

The present study addressed several methodological limitations seen in earlier DRL surveys by restricting analysis to single-phase, standard-range examinations and excluding multiphase, extended-range, trauma, and interventional scans. Standardization using an adult weight range of 50–90 kg reduced variability in body habitus and improved comparability across centers, in line with ICRP recommendations. High-dose outliers resulting from manual exposure overrides, protocol mislabeling, or non-standard acquisitions were excluded to improve data reliability.

The comparison with international DRLs should be interpreted cautiously because protocol definitions, patient selection criteria, scan ranges, contrast practices, and standardization methods vary widely across studies. Therefore, the results should not be interpreted as a simple high-low ranking across countries. Rather, the comparison provides contextual benchmarking. In this study, abdomen–pelvis DRLs were broadly consistent with international reports, whereas head and chest DRLs were slightly higher, indicating opportunities for protocol refinement as mentioned in Table 5.

This study also addresses the need for improved local dose governance within EHS. Local DRLs are particularly important in a multi-hospital system because they enable facility-specific benchmarking, identify outlier practices, and support targeted corrective action. They also provide a practical foundation for future integration with PACS-based dose monitoring systems and annual review processes.

Overall, this study provides a useful framework for structured CT dose management across EHS hospitals. The observed variability confirms that DRLs should be used as tools to identify optimization opportunities rather than as fixed dose limits. Sustainable improvement will require continuous audits, protocol standardization, targeted training, and integration with dose-monitoring systems. These findings contribute to ongoing UAE efforts to update national DRLs and strengthen radiation safety practice.

## 5. Conclusions

This study establishes a consistent, multicenter, standardized framework of Local Diagnostic Reference Levels (DRLs) for common adult CT examinations across Emirates Health Services hospitals, providing an operational baseline for structured dose optimization. Substantial inter-hospital variability highlights that DRLs should be used as tools to identify opportunities for optimization rather than mandate uniform dose limits. While many protocols align with international benchmarks, higher DRLs for chest and head CT examinations indicate the need for continued protocol harmonization, scan-length standardization, and targeted staff training. These local DRLs support ongoing national efforts to update UAE NDRLs, enhance patient safety, standardize CT imaging practice, and maintain diagnostic adequacy.

## Figures and Tables

**Table 1:** CT Scanner Characteristics at Participating EHS Hospitals.

Hospital	Manufacturer	Scanner Model	Company Location	Slice	Installation Year
Fujairah Hospital	GE Healthcare	Discovery HD 750	Chicago, IL, USA	64	2015
Dibba Hospital	GE Healthcare	Revolution	Chicago, IL, USA	128	2015
Masafi Hospital	GE Healthcare	Brivo CT385	Chicago, IL, USA	16	2020
Shaam Hospital	GE Healthcare	Revolution Maxima	Chicago, IL, USA	64	2022
Abdulla Bin Omran Hospital	GE Healthcare	Optima CT660 SGT1700	Chicago, IL, USA	64	2018
Umm Al Quwain Hospital	GE Healthcare	Revolution	Chicago, IL, USA	128	2015
Al Dhaid Hospital	GE Healthcare	Revolution	Chicago, IL, USA	128	2015

**Table 2:** Patient Demographics and Acquisition Parameters by CT Examination Type Values are presented as mean ± SD.

Examination	Age (Years)	Height (cm)	Weight (kg)	BMI (kg·m^−2^)	Tube Voltage (kVp)	mAs	Rotation Time (s)	Pitch
Chest, without contrast	54.5 ± 19.9 (20–100)	163.6 ± 9.2 (135–198)	72.4 ± 12.4 (50–97)	27.1 ± 4.8 (17.4–40.7)	119.6 ± 3.1	117.5 ± 63.7	0.50 ± 0.10	1.00 ± 0.20
Chest, with contrast	37.8 ± 15.2 (20–78)	164.8 ± 7.3 (151–188)	72.9 ± 9.5 (55–90)	26.9 ± 3.7 (20.2–36.4)	117.1 ± 7.1	90.5 ± 46.4	0.60 ± 0.00	1.05 ± 0.15
Head, without contrast	47.7 ± 18.3 (18–99)	162.9 ± 8.6 (136–187)	71.8 ± 11.3 (50–97)	27.1 ± 4.3 (17.0–39.4)	120.1 ± 1.4	165.9 ± 53.9	0.75 ± 0.21	0.50 ± 0.04
Abdomen–pelvis, without contrast	41.8 ± 15.7 (18–84)	163.0 ± 9.9 (100–187)	72.7 ± 11.7 (50–97)	27.6 ± 6.3 (17.7–86.0)	119.7 ± 2.4	131.9 ± 87.4	0.70 ± 0.14	1.17 ± 0.19
Abdomen–pelvis, with contrast	43.9 ± 17.4 (18–93)	163.4 ± 14.5 (116–184)	71.0 ± 9.7 (51–90)	26.9 ± 3.7 (18.2–36.4)	119.3 ± 3.6	149.4 ± 108.4	0.72 ± 0.09	1.17 ± 0.19
Chest–abdomen–pelvis	39.5 ± 16.9 (18–92)	167.3 ± 8.4 (153–188)	73.9 ± 11.9 (53–96.7)	26.5 ± 4.8 (17.1–36.0)	116.1 ± 8.0	155.5 ± 113.5	0.73 ± 0.18	0.98 ± 0.00

Table note: Values are presented as mean ± SD, with range in parentheses. BMI = body mass index.

**Table 3:** CT Dose Indices and Technical Parameters by Examination Type Values are mean ± SD.

Examination	CTDIvol (mGy)	DLP (mGy·cm)
Chest, without contrast	10.0 ± 5.9 (1.2–63.0)	356.8 ± 195.9 (46.0–1179.0)
Chest, with contrast	39.8 ± 47.6 (5.0–308.6)	923.3 ± 519.1 (149.9–2441.1)
Head, without contrast	51.3 ± 9.4 (31.6–101.7)	1000.9 ± 245.2 (546.7–1755.0)
Abdomen–pelvis, without contrast	10.4 ± 8.0 (2.2–90.5)	520.2 ± 283.4 (113.2–1719.2)
Abdomen–pelvis, with contrast	28.4 ± 22.3 (2.5–152.4)	1050.1 ± 716.9 (76.4–4857.7)
Chest–abdomen–pelvis	10.9 ± 5.1 (5.2–23.8)	1094.7 ± 829.0 (313.3–2868.3)

**Table 4:** Median and 75th Percentile Dose Metrics (Proposed Local DRLs).

CT Protocol	CTDIvol		DLP	
	Median (mGy)	75th Percentile (mGy)	Median (mGy·cm)	75th Percentile (mGy·cm)
Chest, without contrast	8.1	12.0	301.1	425.0
Chest, with contrast	29.6	40.6	966.8	1238.0
Head, without contrast	56.9	58.2	1014.3	1179.6
Abdomen–pelvis, without contrast	8.2	11.8	433.0	637.2
Abdomen–pelvis, with contrast	19.2	43.5	901.6	1269.9
Chest–abdomen–pelvis	9.5	15.1	773.3	1411.5

**Table 5:** Comparison of Local Diagnostic Reference Levels from the Present Study with Selected International Studies.

Country	Examination	CT Dose Quantities			
		CTDI_VOL_ (mGy)		DLP (mGy·cm)	
		Median	75th Percentile	Median	75th Percentile
Present Study UAE (2024)	Head, without contrast	56.9	58.2	1014.3	1179.6
	Chest, without contrast	8.1	12.0	301.1	425.0
	Chest, with contrast	29.6	40.6	966.8	1238.0
	Abdomen–pelvis, without contrast	8.2	11.8	433.0	637.2
	Abdomen–pelvis, with contrast	19.2	43.5	901.6	1269.9
	Chest–abdomen–pelvis	9.5	15.1	773.3	1411.5
California (2015) [16]	Head, without contrast	50	62	960	1300
	Chest, without contrast	12	17	550	830
Addis Ababa (2023) [17]	Head, without contrast	51	53	1055	1307
	Chest, without contrast	11	14	517	575
	Abdomen–pelvis, without contrast	11	13	548	932
Turkey (2020) [18]	Head, without contrast	53	59.1	988	1129
	Chest, without contrast	8.3	10.8	314	416
Saudi Arabia (2022) [19]	Head, without contrast	-	59	-	1079
	Chest, without contrast	-	6	-	205
Malaysia (2017) [20]	Head, without contrast	-	46.8	-	1050
	Chest, without contrast	-	21.3	-	420
Singapore (2017) [21]	Head, without contrast	-	51	1052	1079
	Chest, without contrast	-	7	291	322
	Abdomen–pelvis, without contrast	-	12	761	985

## Data Availability

The datasets generated or analyzed during the study are available from the corresponding author on reasonable request.
